# Superior vena cava syndrome: endovascular management

**DOI:** 10.1590/1677-5449.180062

**Published:** 2019-09-24

**Authors:** Walter Kegham Karakhanian, Walter Zavem Karakhanian, Sergio Quilici Belczak

**Affiliations:** 1 Faculdade de Ciências Médicas da Santa Casa de São Paulo – FCMSCSP, Departamento de Cirurgia, Disciplina de Cirurgia Vascular e Endovascular, São Paulo, SP, Brasil

**Keywords:** endovascular procedures, vascular surgical procedures, vena cava, superior vena cava syndrome, procedimentos endovasculares, procedimentos cirúrgicos vasculares, veia cava, síndrome da veia cava superior

## Abstract

**Background:**

The objective of management of superior vena cava syndrome (SVCS) is to promptly alleviate the uncomfortable symptoms. Conventional approaches do not always achieve results as rapidly as endovascular management with stent placement.

**Objectives:**

To report the experience with endovascular management of SVCS of a Vascular and Endovascular Surgery Service at a Brazilian university hospital.

**Methods:**

Symptomatic type III SVCS cases were managed with angioplasty and stent placement in 28 patients aged from 37 to 68 years, between 2002 and 2012. The etiology of SVCS was lung or thoracic cancer in 18 patients, while occlusion of the vein for prolonged use of catheters was the cause in the other 10 cases.

**Results:**

Superior vena cava occlusion repair was not possible in one oligosymptomatic patient with a very severe lesion. Technical success was achieved in 96.4%. There were two deaths, one due to pulmonary embolism, 24 hours after a successful procedure, and the other due to compression of the airways by tumor mass some hours after the procedure. Clinical success was achieved in all cases of technical success, including one patient who died suddenly, after total regression of SVCS symptoms. Symptoms disappeared 24 hours and 48 hours after management in16 and 8 patients respectively; improvement was slower but progressive after 48 hours in the remaining patients.

**Conclusions:**

Endovascular stent placement was effective for management of SVCS, with good technical and clinical success rates and provided prompt relief from symptoms.

## Resumo

**Contexto:**

O tratamento da síndrome da veia cava superior (SVCS) visa aliviar, rapidamente, os sintomas desconfortáveis. Os resultados das abordagens convencionais nem sempre são tão rápidos quanto os do tratamento endovascular com colocação de stent.

**Objetivos:**

Relatar a experiência de um Serviço de Cirurgia Vascular e Endovascular de hospital universitário brasileiro com o tratamento endovascular da SVCS.

**Métodos:**

Vinte e oito pacientes com SVCS sintomática tipo III foram tratados com angioplastia e colocação de stent, entre 2002 e 2012. A idade variou entre 37 e 68 anos. A SVCS resultou de doença neoplásica pulmonar ou torácica em 18 pacientes e de oclusão da veia por uso prolongado de cateteres em 10 pacientes.

**Resultados:**

Não foi possível reparo da oclusão da veia em um paciente oligossintomático com lesão bastante grave. O sucesso técnico foi de 96,4%. Houve duas mortes, sendo uma por embolia pulmonar 24 horas após procedimento bem-sucedido e outra por compressão das vias aéreas por massa tumoral algumas horas após o procedimento. Houve sucesso clínico em todos os casos de sucesso técnico, incluindo o paciente que faleceu de repente após ter apresentado regressão total dos sintomas. Os sintomas desapareceram em 24 e 48 horas após o tratamento em, respectivamente, 16 e oito pacientes. A melhora foi mais lenta, embora progressiva, após 48 horas nos demais pacientes.

**Conclusões:**

A colocação de stent endovascular no tratamento da SVCS foi eficaz, com boas taxas de sucesso técnico e clínico e alívio mais rápido dos sintomas.

## INTRODUCTION

Superior vena cava syndrome (SVCS) is an uncommon clinical condition^1^ and the intensity of clinical manifestations depends on the degree of occlusion or stenosis of the vena cava and on development of collateral circulation, mainly through the azygos vein.

In most cases (85%) it occurs in the presence of malignant diseases, particularly bronchogenic carcinomas, lymphomas, and metastatic tumors.^2^ In about 15% of cases, the cause of SVCS is benign: compression by mediastinal fibrosis or thoracic aortic aneurysm and thrombosis, secondary to use of catheters or pacemaker electrodes or to infusion of chemotherapeutic drugs or parenteral feeding.^3^ It is estimated that 15% of patients with lung cancer and 5% to 20% of those with malignant neoplasia of the thoracic cavity develop SVCS.^4^^,^ 5

The clinical findings classically described include facial, periorbital, cervical, and upper limb edemas followed by venous dilatations of the anterior thoracic wall, characterizing collateral circulation. Although it is usually not life threatening, SVCS is frequently associated with uncomfortable symptoms, such as dyspnea, dysphagia, and cognitive alterations, and intracranial venous hypertension can result in coma.^6^

Endovascular management with stenting has proven effective for alleviation of the aforementioned symptoms. The objective of this study is to report the experience with endovascular management of SVCS of a Vascular and Endovascular Surgery Service at a Brazilian university hospital and to determine technical and clinical success rates.

## METHODS

### Design

This medical record review conducted at the Department of Surgery at the Santa Casa de Misericordia de São Paulo was not submitted for Ethics Committee approval.

Patients’ clinical severity is based on the intensity of facial, neurological, and respiratory symptoms, which are related to the degree of superior vena cava stenosis (obstruction) and the direction of flow through the azygos vein. Stanford^7^ has classified these anatomic and physiologic aspects into four types: (I) stenosis < 90% with a patent azygos vein; (II) stenosis between 90%-100% with anterograde azygos vein flow; (III) stenosis between 90%-100% with retrograde azygos vein flow; (IV) occlusion of both superior vena cava and azygos vein. All patients included in this study were treated for Stanford type III.

### Patients

The study included data from all consecutive symptomatic patients with type III SVCS (stenosis between 90%-100% with retrograde azygos vein flow),^7^ referred by the Oncology Service as urgent medical cases between 2002 and 2012 and managed with angioplasty and stenting, as described below.

All patients were followed up for 90 days to assess their response to endovascular management in terms of alleviation of symptoms directly related to occlusion of the superior vena cava, recurrence, and complications caused by endovascular procedures.

### Technical details

From 2002 to 2005, procedures started with a phlebographic study conducted by puncture of superficial veins in both upper limbs and inferior cavography to assess collateral circulation, the extension of the lesion and involvement of subclavian and brachycephalic veins, the distal segment of the superior vena cava next to the right atrium, and dominance of the jugular vein. In some cases, with a very swollen arm making puncture of a vein difficult, the phlebographic study was carried out using a right common femoral approach or via right and left jugular veins. Heparin 5000 IU (1 ml) was injected into the peripheral vein after puncture.

From 2006, careful computed tomography of the thorax replaced phlebography to provide similar information for planning endovascular management. The stenosis/occlusion was negotiated with a 0.035 hydrophilic guidewire (Terumo or “Roadrunner” by Cook) and an MP 5 FR catheter as support. After guidewire insertion, we analyzed the extent of the lesion, while planning the place where the stent(s) would be anchored: the proximal part of the superior vena cava, or one of the brachiocephalic veins, or the jugular vein. Some situations required the hydrophilic guidewire to be replaced with a more rigid guidewire (Amplatz Cook) to better support navigation of the stents. Lesion length and vein diameter were assessed with the aid of a centimeter-scale pigtail catheter (Cook).

**Figure 1:**
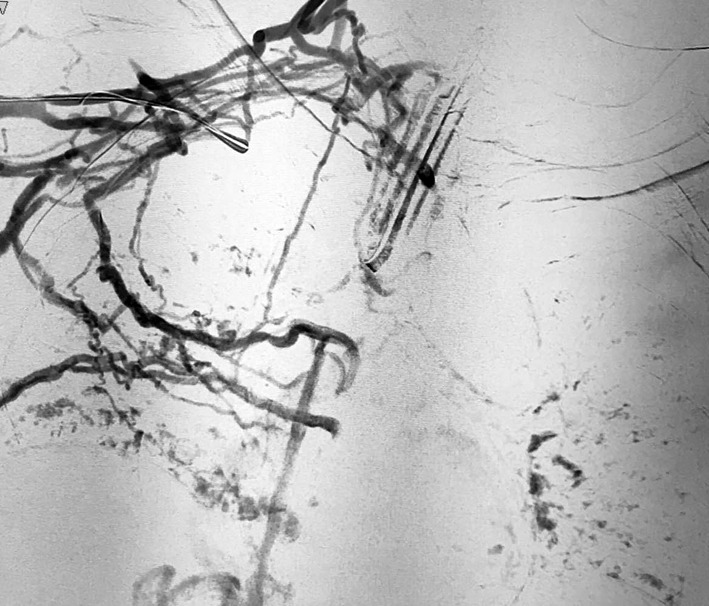
Occlusion of superior vena cava and rich collateral circulation shown by phlebography in a patient with SVCS.

In cases of sub-occlusions or occlusions (Figure 1), we performed pre-dilatation with an 8 mm x 40 mm or an 8 mm x 60 mm balloon, already with an idea of the extension of the lesion and the diameter of the vena cava. The proximal stent was placed and released first. When necessary, other stents were deployed to provide overlapping protection of the entire superior vena cava extension up to the beginning of the right atrium.

**Figure 2:**
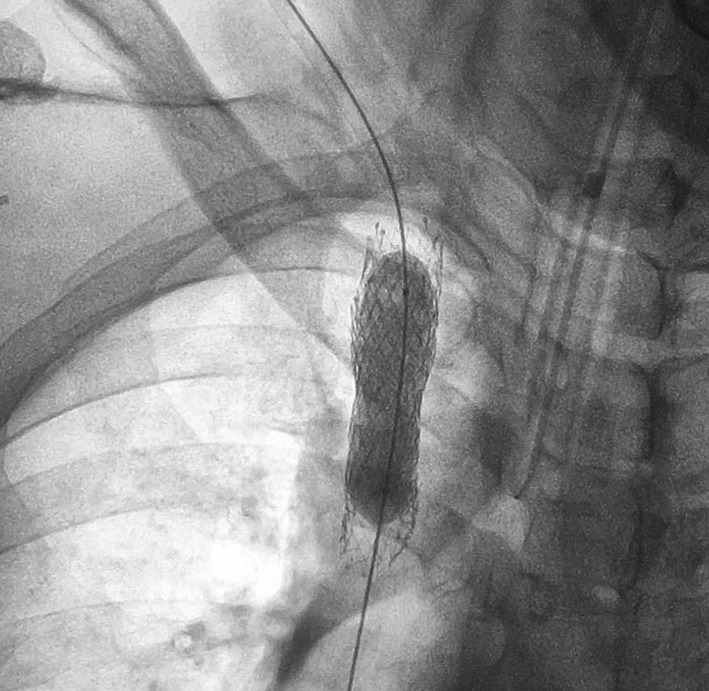
Ballooning of Sioxx stent placed at obstruction site in superior vena cava.

Self-expanding stents with diameters between 10 mm and 24 mm were the preferred choice. Wallstents (Boston Scientific) were used in most cases. A sinus stent (Optimed) was chosen for one case and a Sioxx stent (Scitech) was used in another (Figure 2). Stent lengths varied from 40 to 80 mm, depending on the area of obstruction. More than one stent was needed in two cases.

In one patient, a fibrinolytic (20 mg rtPA in bolus) was used concomitantly to prevent pulmonary embolism. Post-dilatation with compatible balloons (16 mm or 18 mm in diameter) was performed at the point of greatest constriction. Balloon-expandable stents were not used in any cases.

All patients were put on dual platelet inhibition only, except for one who was put on anticoagulants as well.

### Data collection

For this descriptive study, data taken from medical records included gender and age of the patients, etiology of the SVCS, technical and clinical success of the procedures, time (in hours) to clinical success after the procedure, and information on recurrences, complications, and deaths. Data are presented as absolute and percentual frequencies, except for age, which is expressed as means.

### Outcomes

Outcomes included the technical and clinical success of the procedures. Technical success was defined as complete patency of the treated segment, with good contrast flow velocity. Clinical success was defined as reduction of the symptoms of facial and cervical edema, dyspnea, and cognitive alterations resulting from cerebral edema, when present.

## RESULTS

Table 1 shows data from the medical records of 28 patients with symptomatic type III SVCS managed with angioplasty and stenting.

**Table 1:** Data from medical records of 28 patients with symptomatic type III superior vena cava syndrome managed with endovascular procedures.

Age (mean/range)	52.5/37-68 years old
Gender (n/%)	
Male	26/92.9%
Female	2/7.1%
Etiology of SVCS (n/%)	
Malignant disease*	18/64.3%
Prolonged use of catheters	10/35.7%
Outcomes (n/%)	
Technical success	27/96.4%
Clinical success	27/96.4%
- 24 hours after procedure	16/57.1%
- 48 hours after procedure	8/28.6%
- more than 48 hours after procedure	3/10.7%

(*) 16 bronchogenic carcinomas and 2 small cell lung carcinomas

Technical success was judged to have been achieved in 27 patients (96.4%). Superior vena cava occlusion repair was not possible in one oligosymptomatic patient, probably because of the severity of the lesion. One patient died 24 hours after a successful procedure, probably due to pulmonary embolism. Another died some hours after the procedure after compression of the airways by tumor mass.

**Figure 3:**
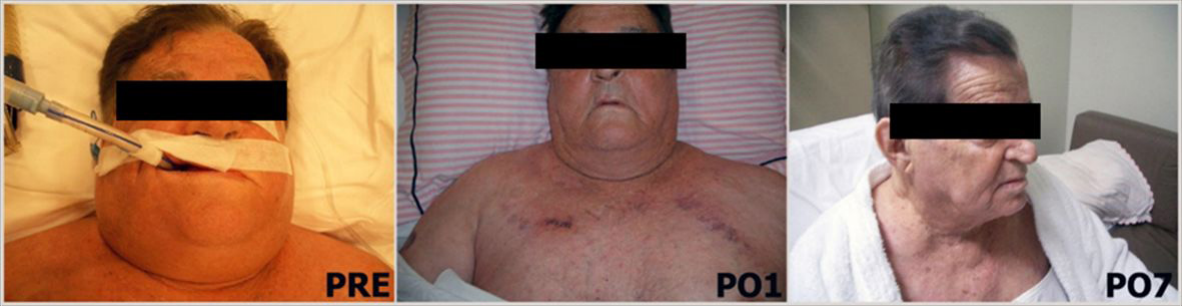
73-year-old patient before intervention (PRE); and one (PO1); and seven (PO7) days after intervention.

Clinical success followed technical success in all cases (Figure 3), including the patient who died suddenly after presenting total regression of the SVCS symptoms, which had been very evident before the procedure. Symptoms disappeared 24 hours after management for 16 patients (59.2%). Equivalent improvement was observed at 48 hours after the procedure in 8 patients (29.6%). Relief from symptoms was slower but progressive after 48 hours in the remaining 3 patients.

## DISCUSSION

Since SVCS is a rare diagnosis and considering our relevant sample of 28 symptomatic patients managed with angioplasty and stenting over a period of 10 years, it is important to report our experience of the technical and clinical success of this management approach. To accomplish this, we reviewed the medical records of symptomatic patients with Standford type III SVCS only.

Fifty years ago, the main etiology of SVCS was attributed to infectious causes, particularly syphilitic aneurysm of the thoracic aorta or tuberculosis, but this has been replaced by malignant diseases, especially lung tumors.^8^ Currently, benign causes resulting from increasing use of intravascular catheters and pacemaker electrodes are responsible for at least 35% of cases, and this is an ascending curve.^9^ In fact, SVCS in 10 of the 28 patients (35.7%) in our sample was derived from benign causes, more frequently in more recent years.

SVCS is a clinical diagnosis confirmed by computed tomography (CT) with contrast medium, which also enables the assessment of the extension of stenosis/occlusion, without the need for phlebography except during endovascular management. At the beginning of our experience, phlebography via upper limbs with simultaneous injection of contrast was routine, since it enabled assessment of the involvement of subclavian and brachycephalic veins. With the progressive improvements in CT, we abandoned this technique, reducing the volume of contrast as well as the number of accesses. Currently, most patients can be treated with access via the right common femoral vein, with lower complication rates. Magnetic resonance has not been widely used and is reserved for patients intolerant of iodinated contrast medium.^10^ PET scans are useful when planning an area to be irradiated.^11^

Endovascular therapy is indicated when conservative management fails to improve the symptoms or is followed by symptom progression.^5^^,^ 12 The hydration needed during administration of chemotherapy drugs can exacerbate symptomatology, and in such circumstances preemptive stent implantation can avoid decompensation resulting from excessive administration of liquids. This was the case with one of our patients: his Oncologist had requested recanalization to prevent decompensation. Technical success was not achieved in this case and phlebography showed occlusion of the superior vena cava, but with sufficient dilatation of the azygos vein to enable satisfactory venous return.

In some situations, SVCS becomes a medical emergency with poor prognosis, particularly if cerebral edema occurs or if laryngeal edema compromises the patency airways.^4^

Most of our patients were treated urgently because they presented with very evident or even dramatic symptoms.

In successful endovascular management, relief from symptoms is observed 24 to 48 hours after the procedures in about 68%-100% of the patients.^13^ In cases of malignant diseases, recurrence is reported in up to 20%^5^^,^ 14 and is caused by disease progression or sometimes by displacement of the stent.^5^ Evident clinical improvement in 24 to 48 hours was observed in 90% of our patients.

Before the availability of endovascular management, relief from symptoms caused by venous obstruction was possible with radiotherapy and chemotherapy^15^^-^ 17 and exceptionally with open surgical intervention. High doses of corticoids, diuretics, and anticoagulants have also been administered, but with uncertain results.^18^

Chemotherapy and radiotherapy effectively reduce the tumor by about 60%,^19^ and symptoms improve in 90% of the cases. However, these events are only observed 3 to 4 weeks after procedures, limiting the utility of these options for management of emergencies. Furthermore, recurrence rates after conservative treatment vary from 10 to 50%.^20^^,^ 21 Surgical reconstruction with grafts was reserved for cases of failure with conservative treatment.^22^

With advances in interventional techniques, new therapeutic methods were proposed to alleviate the symptoms produced by superior vena cava occlusion. Vein angioplasty with balloons is one possibility, but results are disappointing.^23^ Since stenting for SVCS was first reported, this procedure has been carried out with immediate relief and/or complete resolution of symptoms.^24^^,^ 25

The most frequently used stents are Palmaz (Cordis), Wallstent (Boston Scientific), and Gianturco-Z-Stent (Cook). Although there is a lack of studies comparing these different devices, there is an international tendency to use the Wallstent, since it is self-expanding and is not compressed by external forces. These stents are measured at the time of intervention and it is worth emphasizing that Wallstents can shorten by up to 30% and that the diameter should be estimated at 120 to 150% of the diameter of the superior vena cava.^21^ Since large stents were unavailable when we treated the first patients, their diameters were undersized, and even though there were no complications because of this reason, we currently recommend stents with 18 to 22 mm diameters.

There are situations when compression or possible thrombosis compromise the whole vena cava as well as the brachiocephalic veins, so there is no adequate proximal segment for stent anchoring. Technical options in such cases include kissing stents (double stents) or choosing one of the brachiocephalic veins (generally the dominant one) to implant a single stent. In common with other authors, we believe that the single stent implantation technique is superior to kissing stents, since the complication rates are lower.^26^ Although we avoid extension of the distal stent portion into the right atrium whenever possible, we were obliged to do this in three of the patients in our sample. Since there is no consistent evidence on use of covered stents, we preferred not to use them because we did not find it necessary.

Complication rates are reported at about 3 to 7%, including bleeding, infection, migration, occlusion, pulmonary embolism, and, rarely, perforation.^27^ Except for two deaths, we had no severe complications. Some hematomas occurred at the puncture site, but without clinical significance or need for surgical intervention.

Use of anticoagulation and antiplatelet treatments after stent deployment in the superior vena cava is still controversial and there is no consensus.^28^^,^ 29 In the absence of any contraindications, we mainly kept our patients on antiplatelet therapy.

## CONCLUSION

In our practice, endovascular stent implantation has been effective for management of symptomatic SVCS type III, with good technical and clinical success rates, providing prompt relief from symptoms.
